# Safety and effectiveness of eculizumab for adult patients with atypical hemolytic–uremic syndrome in Japan: interim analysis of post-marketing surveillance

**DOI:** 10.1007/s10157-018-1609-8

**Published:** 2018-06-29

**Authors:** Hideki Kato, Yoshitaka Miyakawa, Yoshihiko Hidaka, Norimitsu Inoue, Shuichi Ito, Shoji Kagami, Shinya Kaname, Masanori Matsumoto, Masashi Mizuno, Takahisa Matsuda, Akihiko Shimono, Shoichi Maruyama, Yoshihiro Fujimura, Masaomi Nangaku, Hirokazu Okada

**Affiliations:** 1 Division of Nephrology and Endocrinology, The University of Tokyo, 7-3-1, Hongo, Bunkyo-ku, Tokyo, 113-8655 Japan; 2 Department of General Internal Medicine, Thrombosis and Hemostasis Center, Saitama Medical University, 38 Moroyama, Iruma-gun, Saitama, 350-0495 Japan; 3 Clinical Division of Pediatrics, Shinshu University Hospital, 3-1-1 Asahi, Matsumoto, Nagano 390-8621 Japan; 4 Department of Tumor Immunology, Osaka International Cancer Institute, 3-1-69 Otemae, Chuo-ku, Osaka, 541-8567 Japan; 5 Department of Pediatrics, Graduate School of Medicine, Yokohama City University, 3-9 Fukuura, Kanasawa-ku, Yokohama, Kanagawa 236-0004 Japan; 6 Department of Pediatrics, Institute of Health Biosciences, The University of Tokushima Graduate School, Kuramoto cho 3-chome, Tokushima, 770-8503 Japan; 7 Department of Nephrology and Rheumatology, Kyorin University School of Medicine, 6-20-2 Shinkawa, Mitaka, Tokyo 181-8611 Japan; 8 Department of Blood Transfusion Medicine, Nara Medical University, 840 Shijyo-cho, Kashihara, Nara 634-8522 Japan; 9 Renal Replacement Therapy, Division of Nephrology, Nagoya University Graduate School of Medicine, 65 Tsurumai-cho, Showa-ku, Nagoya, Aichi 466-8550 Japan; 10 Alexion Pharma GK, 1-18-14 Ebisu, Shibuya-ku, Tokyo, 150-0013 Japan; 11 Department of Nephrology, Nagoya University Graduate School of Medicine, 65 Tsurumai-cho, Showa-ku, Nagoya, Aichi 466-8550 Japan; 12 Japanese Red Cross Kinki Block Blood Center, 7-5-17, Saitoasagi, Ibaraki, Osaka 567-0085 Japan; 13 Department of Nephrology, Saitama Medical University, 38 Moroyama, Iruma-gun, Saitama, 350-0495 Japan

**Keywords:** Atypical hemolytic–uremic syndrome, Post-marketing surveillance, Complement, C5 inhibitor, Eculizumab

## Abstract

**Background:**

Eculizumab has been available for the treatment of atypical hemolytic–uremic syndrome (aHUS) in Japan since 2013. To assess safety and effectiveness of eculizumab in adult aHUS patients in the real-life setting, we performed interim analysis of a post-marketing surveillance mandated by Japanese regulations.

**Methods:**

This study enrolled any patient who was diagnosed with TMA excluding Shiga toxin-producing *Escherichia coli*-HUS or thrombotic thrombocytopenic purpura based on Japanese clinical guide published in 2013 as inclusion criteria and treated with eculizumab. Although the term aHUS was redefined to denote only complement-mediated HUS in the guide revised in 2016, the patients with TMA caused by other causes (secondary TMA) were included. Patient outcomes and safety were evaluated at 6 months, 12 months, and annually thereafter.

**Results:**

Thirty-three patients with aHUS and 27 patients with secondary TMA were enrolled. Median treatment duration of aHUS was 24weeks. Complement genes variants were detected in 11 of 18 patients with aHUS (61.1%). Among the 29 aHUS patients with available baseline data, platelet count (PLT), lactic dehydrogenase and serum creatinine (SCr) improved within 1-month after eculizumab initiation. TMA event-free status, complete TMA response, PLT normalization, and SCr decrease were achieved in 67.9% (19/28), 27.8% (5/18), 56.5% (13/23), and 57.1% (16/28) of patients, respectively. Thirty-three and 11 adverse reactions were observed in patients with aHUS (13/33 patients) and secondary TMA (6/27 patients), respectively.

**Conclusions:**

This interim analysis confirmed the acceptable safety profile and effectiveness of eculizumab for Japanese adult aHUS patients in real-world settings.

**Electronic supplementary material:**

The online version of this article (10.1007/s10157-018-1609-8) contains supplementary material, which is available to authorized users.

## Introduction

Atypical hemolytic–uremic syndrome (aHUS), a form of thrombotic microangiopathy (TMA), is a rare disease characterized by the triad of microangiopathic hemolytic anemia (MAHA), thrombocytopenia, and acute kidney injury (AKI) [1–3]. Mutations of complement genes are found in approximately 40–60% of patients; however, the penetrance of the disease is approximately 50%. Since age at onset and clinical severity are variable among individuals, aHUS affects patients of all ages [4, 5].

Outcomes are poor even when aHUS is treated with plasma therapy, and patients are at high risk of end-stage renal-disease (ESRD) and death [4, 5]. An observational study of 214 patients with aHUS in France showed that 56% of adults progressed to ESRD or death within 1 year of follow-up [4]. In an Italian study, 67% of adults with aHUS required dialysis or died within 3 years, when treated with plasma therapy [5].

In Japan, the diagnostic criteria for aHUS were defined in the 2013 clinical guides [6, 7] of the Japanese Society of Nephrology and Japan Pediatric Society. These criteria were then revised in 2015 clinical guides, to account for recent findings and the emerging international consensus on aHUS [2, 3]. The 2013 criteria [6, 7] defined aHUS broadly as TMAs not including Shiga toxin–producing *Escherichia coli* (STEC)-HUS or thrombotic thrombocytopenic purpura (TTP). We refer to this as the “broad aHUS definition”. In the 2015 updated clinical guide, aHUS (complement-mediated HUS) was defined as TMA satisfying the 2013 criteria [6, 7] but not including “secondary TMA”, i.e., TMAs associated with transplantation, infection, drugs, autoimmune diseases, malignant tumors, or metabolic disorders [2, 3]. Accordingly, aHUS (complement-mediated HUS) was defined by TMAs caused by a congenital or acquired complement regulation abnormality or, in patients without a known relevant genetic mutation, a clinical TMA manifestation indicative of aHUS that cannot be classified as STEC-HUS, TTP, or secondary TMA. The narrow definition of aHUS is known as complement-mediated HUS [2, 3] or primary aHUS [8], which we refer to as “aHUS” in this report [6, 7].

In September 2013, the recombinant humanized monoclonal antibody, Eculizumab (Soliris^®^, Alexion Pharmaceuticals), was approved for treatment of aHUS in Japan [9] on the basis of the results of clinical studies [10, 11]. Eculizumab binds complement component C5 and prevents its cleavage by C5 convertases. Data on its safety and effectiveness in patients with aHUS are limited; therefore, the Ministry of Health, Labour and Welfare (MHLW) of Japan requested Alexion Pharma GK to monitor all aHUS patients treated with eculizumab, as a condition for approval of eculizumab. Thus, regulatory-mandated post-marketing surveillance (PMS) in Japan began to assess the long-term safety and effectiveness of eculizumab for aHUS patients treated in clinical practice. As a consequence of the changes in definition of aHUS in Japan, the inclusion criteria changed over the time of the study. Herein, we conducted an interim analysis if the PMS to assess the safety and effectiveness of eculizumab for treatment of aHUS in Japanese adult patients.

## Methods

### Study design and patients

The PMS is a Japanese government mandated regulatory observational study to evaluate safety and effectiveness of eculizumab in patients with aHUS in a real-life setting in Japan. This study was conducted in accordance with good post-marketing study practice (GPSP) for drugs (MHLW Ministerial Ordinance No. 171 of 2004), and the requirements of ethical approval and informed consent from individual patients were waived because of the mandatory nature of the study. PMS started in September 2013, and this interim analysis includes data collected up to March 15, 2017.

Adult patients older than or equal to 18 years who received an aHUS diagnosis based on the contemporaneous Japanese diagnostic guide and received at least 1 dose of eculizumab were included. The definition and diagnostic criteria for aHUS have evolved over time in Japan, and the inclusion criteria have thus changed during the study. In the 2013 criteria, aHUS was diagnosed when MAHA, thrombocytopenia, and AKI were present, after excluding STEC-HUS and TTP [6, 7]. In 2015, the criteria evolved to exclude TMAs associated with transplantation, infection, drugs, autoimmune diseases, malignant tumors, or metabolic disorders [2, 3]. TTP was excluded if a disintegrin-like and metalloproteinase with thrombospondin type 1 motifs 13(ADAMTS13) activity was < 5–10%. MAHA was defined as a hemoglobin level of < 10 g/dL and thrombocytopenia as a platelet count (PLT) of < 15 × 10^4^/µL. AKI was defined using the Kidney Disease Improving Global Outcomes guideline [12].

Characteristics at the initiation of eculizumab treatment (baseline) were recorded and patient outcomes and safety were evaluated at follow-up examinations at 6 months, 12 months, and annually thereafter. Data on patient demographics (age, sex, body weight, and family history of aHUS) and disease characteristics (genetic mutations or polymorphisms, past treatment, laboratory findings) at the start of eculizumab administration was evaluated. Genetic information on complement genes was also analyzed.

### Treatment

Patients received intravenous eculizumab as decided by the attending physician and patient. The labeled dosing is 900 mg weekly for the first 4 weeks, 1200 mg for the fifth dose, 1 week later, and 1200 mg every 2 weeks thereafter. Anti-meningococcal vaccination is mandatory before the first dose of eculizumab [9]. Data were collected on the duration of eculizumab administration, the regimen used, and the reason for discontinuation.

### Assessments of safety and effectiveness

Adverse events (AEs) and adverse reactions (ARs) of eculizumab were classified according to the Japanese translation of the Medical Dictionary for Regulatory Activities and defined as shown in Supplementary Table 1.

The endpoints of effectiveness were TMA event-free status, complete TMA response, hematologic outcomes, and renal outcomes, as described in Supplementary Table 1. eGFR was calculated using the following formula: 194 × creatinine level-1.094 × age-0.287 (× 0.739, if female) [13]. eGFR is commonly used to describe chronic kidney disease, but was used to evaluate AKI in this study.

### Statistical analysis

Descriptive analysis was performed using median, mean, standard deviation (SD), and range (for continuous variables) and frequency and proportions (for categorical variables). In the safety analysis, the numbers of patients and incidence rates (in person-years) for each event were calculated. In the effectiveness analysis, the numbers and proportions of patients who achieved each of the endpoints of interest during treatment were calculated. Absolute values and changes from baseline in PLT, lactate dehydrogenase (LDH), and eGFR were summarized using descriptive statistics.

Missing data were not imputed, except for body weight at the time of eculizumab administration, which was imputed using the most recent data before administration. Statistical analyses were performed with SAS version 9.1.3 (SAS Institute, Cary, NC). Two-sided *P* values (significance level 0.05) were used in all analyses.

## Results

### Adult patients enrolled in PMS

A total of 60 adult patients who satisfied the “broad aHUS definition” were included in the study: 33 patients with narrowly defined aHUS (complement-mediated HUS) and 27 patients with secondary TMAs. The 33 patients with aHUS were included for safety analysis. Effectiveness was analyzed for 29 patients with aHUS, because 4 patients with aHUS started eculizumab before drug approval and baseline data were not available. In addition, we evaluated outcomes and safety in all 27 patients with secondary TMA.

### Characteristics of patients with aHUS (complement-mediated HUS)

Median age (range) at first eculizumab administration was 58 (20–81) years and median weight (range) was 54.3 (29.1–100) kg. Complement related-genes and autoantibody against complement factor H (CFH) at diagnosis were examined in 18 patients: of these patients, gene mutation, polymorphism or autoantibody was identified in 11 patients (61.1%) (Table 1). Identified variants and allele prevalence are summarized in Supplementary Table 2. No patient had the acquired form of aHUS associated with autoantibodies against CFH.

**Table 1:** Characteristics of aHUS (complement-mediated HUS) patients at the start of eculizumab administration (*n* = 29)

Median age at 1st eculizumab administration (range) *n* = 29	58 (20–81)
Median weight, kg (range)	54.3 (29.1–100)
Female sex, *n* (%)	13 (44.8)/29
Patient reported family history of aHUS, *n* (%)	0 (0)/29
Identified complement gene variant, autoantibody or polymorphism, *n* (%)/examined	11 (61.1)/18
*C3, n* (%)	3 (27.3)/11
*CFB, n (%)*	2 (18.2)/11
*CFH, n* (%)	7 (63.6)/11
*CFHR1*/*3* deletion, *n* (%)	0 (0.0)/11
CFH antibody, *n* (%)	0 (0.0)/11
*CFI, n* (%)	0 (0.0)/11
*MCP, n* (%)	1 (9.1)/11
*DGKE, n* (%)	0 (0.0)/11
*THBD, n (%)*	1 (9.1)/11
Other^a^, *n* (%)	1 (9.1)/11
Two or more variants or polymorphisms^b^	2 (18.2)/11
Median period from 1st TMA symptom to the first administration of eculizumab, (days) median (range)	22.5 (1–963)
Median period from the day of diagnosis to the first administration of eculizumab (days), median (range)	4 (1–88)
Plasma therapy (past 1 year), *n* (%)	18 (62.1)/29
Median days of plasma therapy implementation from TMA symptom closest to the timing of diagnosis to the day before first administration of eculizumab (days), median (range)	5 (0–25)
Dialysis at diagnosis (past 1 year), *n* (%)	17 (58.6)/29
Previous renal transplant, *n* (%)	0 (0.0)/29
Median platelet count, × 10^4^/µl, (range)	5.20 (0.1–21.1)
Platelet count < 150 × 10^4^/µl, *n* (%)	25 (86.2)/29
Median LDH level, U/l (range)	402 (161–3882)
LDH greater than ULN, n (%)	23 (79.3)/29
Median hemoglobin concentration, g/dl (range)	8.2 (5.2–13.5)
Hemoglobin concentration < 10 g/dl	28 (96.6)/29
Schistocytes positive, *n* (%)	8 (88.9)/9
Median serum creatinine level, mg/dl (range)	3.67 (0.9–21.5)
Median eGFR, ml/min/1.73 m^2^ (range)	14.09 (2.1–78.0)
eGFR (ml/min/1.73 m^2^), *n*	29
< 15, *n* (%)	16 (55.2)
15–29, *n* (%)	6 (20.7)
30–44, *n* (%)	5 (17.2)
45–59, *n* (%)	0 (0)
60–89, *n* (%)	2 (6.9)
≥ 90, *n* (%)	0 (0)
Median duration of eculizumab treatment, weeks (range), *n* = 29	24.0 (0–103)
< 1 week, *n* (%)	3 (10.3)
≥ 1, < 4 weeks, *n* (%)	5 (17.2)
≥ 4, < 26 weeks, *n* (%)	8 (27.6)
≥ 26 weeks, *n* (%)	13 (44.8)

*C3* complement component 3, *CFB* complement factor B, *CFH* complement factor H, *CFHR* CFH-related protein, *CFI* complement factor I, *MCP* membrane cofactor protein, *DGKE* diacylglycerol kinase ε, *THBD* thrombomodulin. CFHR1/3 denotes the locus from the CFHR3 to the CFHR1 genes. *TMA* thrombotic microangiopathy, *LDH* lactate dehydrogenase, *ULN* upper limit of normal, *eGFR* estimated glomerular filtration rate

^a^CFHR5 variant p.Pro453Ser

^b^One combination of identified genetic variants was CFB p.Leu9His, CFH p.Glu936Asp, CFHR5 p.Pro453Ser and THBD p.Ala473Val. The other combination was CFB p.Arg32Gln and CFH p.Val62Ile - p.His402Tyr -p.Glu936Asp

Among patients with aHUS, the median time (range) from first TMA occurrence and aHUS diagnosis to the first dose of eculizumab was 22.5 (1–963) days and 4 (1–88) days, respectively (Table 1). Eighteen aHUS patients (62.1%) had received plasma therapy during the previous 1 year, and 17 aHUS patients (58.6%) were receiving dialysis at diagnosis. Prior medical history like liver dysfunction and malignant tumors was reported in 58.6% of patients with aHUS, as shown in Supplementary Table 3.

The median (range) total duration of eculizumab treatment was 24.0 (0–103) weeks. Three patients were treated with eculizumab for < 1 week, five patients for ≥ 1 to < 4 weeks, eight patients for ≥ 4 to < 26 weeks, and 13 patients for ≥ 26 weeks. At the date of data cut-off, 10 adult aHUS patients were continuing eculizumab treatment and 19 aHUS patients had discontinued eculizumab. Reasons for discontinuation of eculizumab were doctor’s judgement (*n* = 8), insufficient response to the treatment (*n* = 5), death (*n* = 4), patient decision and adverse event (*n* = 3 each), and other reasons (*n* = 1) (Supplementary Table 4).

### Effectiveness of eculizumab for aHUS (complement-mediated HUS)

The effectiveness endpoints during eculizumab treatment of 24 weeks (median) for patients with aHUS are shown in Table 2. TMA event-free status was achieved in 19/28 patients (67.9%, 95% CI 47.6–84.1%). Complete TMA response, which was defined as maintenance of hematologic and renal outcomes for 4 weeks, and hematologic normalization were achieved in 5/18 patients (27.8%, 95% CI 9.7–53.5%) and 7/18 patients (38.9%, 95% CI 17.3–64.3%), respectively.

**Table 2:** Endpoint in aHUS (complement-mediated HUS) patients

TMA event-free status, *n*	28
*n* (%)	19 (67.9)
95% CI	47.6–84.1
Complete TMA response, *n*	18
*n* (%)	5 (27.8)
95% CI	9.7–53.5
Hematologic outcome	
Hematologic normalization, *n*	18
*n* (%)	7 (38.9)
95% CI	17.3–64.3
Platelet count normalization, *n*	23
*n* (%)	13 (56.5)
95% CI	34.5–76.8
LDH normalization, *n*	22
*n* (%)	12 (54.5)
95% CI	32.2–75.6
Hemoglobin improvement ≥ 2 g/dl, *n*	28
*n* (%)	14 (50.0)
95% CI	30.6–69.4
Renal outcome	
Serum creatinine level decrease by ≥ 25%, *n*	28
*n* (%)	16 (57.1)
95% CI	37.2–75.5
eGFR improvement by ≥ 15 ml/min/1.73 m^2^, *n*	26
*n* (%)	4 (15.4)
95% CI	4.4–34.9

*CI* confidence interval, *eGFR* estimated glomerular filtration rate, *LDH* lactate dehydrogenase, *TMA* thrombotic microangiopathy

**Fig. 1:**
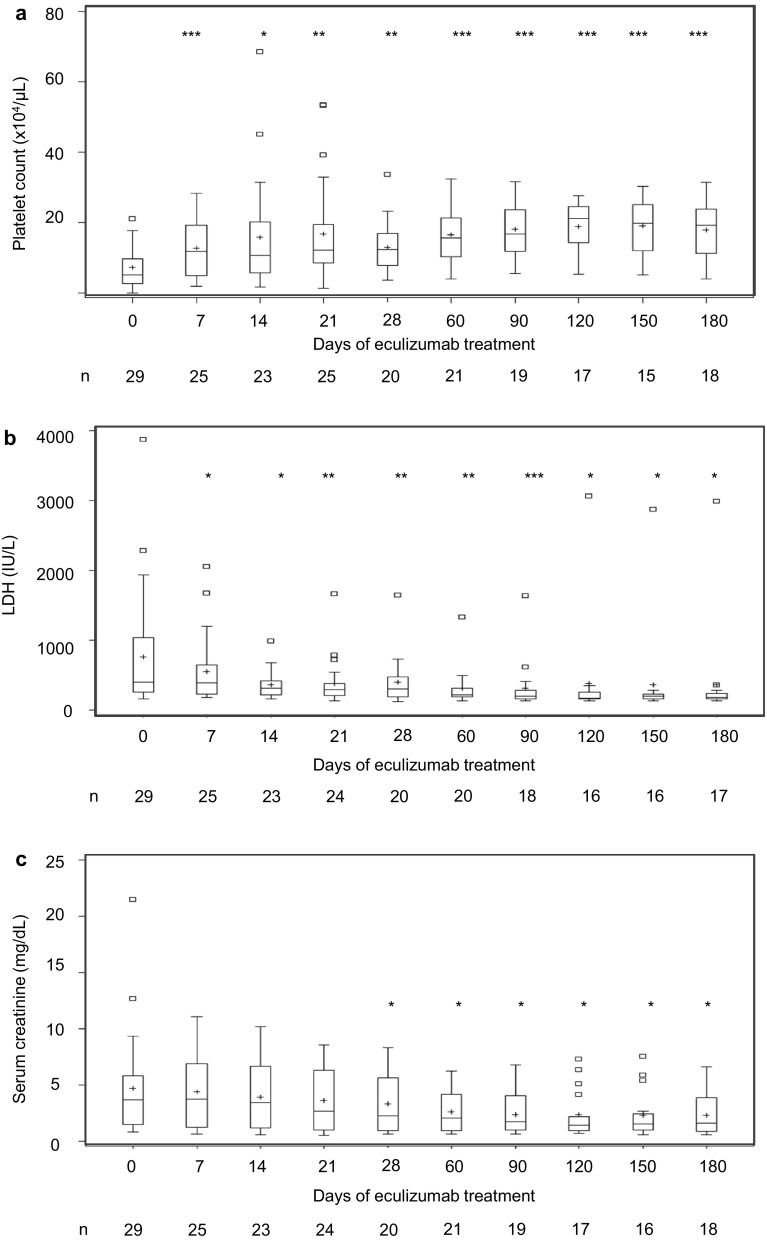
The level of platelet count, LDH and Serum creatinine during eculizumab treatment of aHUS patients. **a** PLT was significantly improved from 1week of eculizumab initiation in aHUS (complement-mediated HUS) patients. Changes from baseline were compared using the paired *t* test. *P < 0.05, **P < 0.01, ***P < 0.001 vs eculizumab initiation. **b** LDH was significantly improved from 1week of eculizumab initiation in aHUS (complement-mediated HUS) patients. Changes from baseline were compared using the paired *t* test. *P < 0.05, **P < 0.01, ***P < 0.001 vs eculizumab initiation. **c** SCr was significantly improved from 4 weeks of eculizumab initiation in aHUS (complement-mediated HUS) patients. Changes from baseline were compared using the paired *t* test. *P < 0.05, vs eculizumab initiation

Median (range) PLT was 5.2 (0.1–21.1) at baseline and 10.7 (1.8–68.7) × 10^4^/µL at 14 days (Fig. 1a, Supplementary Fig. 2). Mean change in PLT from baseline to 14 days was 8.1 ± 15.7 × 10^4^/µL (*P* = 0.021). PLT normalization was achieved in 13/23 patients (56.5%, 95% CI 34.5–76.8%) (Table 2). Median (range) LDH was 402.0 (161–3882) IU/L at baseline and 320.0 (165–988) IU/L at 14 days (Fig. 1b, Supplementary Fig. 2). Mean change in LDH from baseline to 14 days was − 253 ± 429 IU/L (*P* = 0.01). LDH normalization was achieved in 12/22 patients (54.5%, 95% CI 32.2–75.6%) (Table 2). Median (range) SCr was 3.67 (0.9–21.5) mg/dL at baseline, 2.26 (0.7–8.3) mg/dL at 28 days, and 2.10 (0.7–6.3) mg/dL at 60 days (Fig. 1c, Supplementary Fig. 2). The mean change in serum creatinine from baseline to 28 days and from baseline to 60 days was − 1.1 ± 2.0 and − 2.4 ± 4.5 mg/dL (*P* = 0.018 and *P* = 0.024), respectively. A decrease of ≥ 25% in SCr was achieved in 16/28 patients (57.1%, 95% CI 37.2–75.5%) (Table 2). These hematologic and renal outcomes were maintained throughout the treatment period. In addition, dialysis was discontinued in 9 of 17 patients who had required dialysis at baseline. The overall survival of aHUS patients was 88.2%, as shown in Supplementary Fig. 1.

### Safety for aHUS (complement-mediated HUS)

During eculizumab treatment, total exposure time was 16.3 patient-years, and 33 ARs (2.02 per patient-year) were reported in 13 patients during eculizumab treatment (Table 3). ARs reported in 2 or more patients were hypertension (four patients, 0.25 per patient-year), nasopharyngitis, hyperuricemia, and edema (two patients each, 0.12 per patient-year for all); the other 23 ARs occurred in 1 patient each (0.06 per patient-year for all). Neither meningococcal infection nor infusion reaction was reported during eculizumab treatment. The vaccination rate in aHUS patients was 81.8% (27/33). Ten serious ARs were reported in seven patients (0.61 per patient-year); all reactions except for hypertension were not listed on the product label [9]. Of these, pneumonia was the only infection-related serious AR (1 patient). No association with eculizumab was judged in the four patients who died in the study (further described in Supplementary Table 5).

**Table 3:** Treatment-emergent adverse reaction in aHUS (complement-mediated HUS) patients (*n* = 33)

	Adverse reaction	Serious adverse reaction
	Number of cases (/person-year)	Number of cases (/person-year)
Total exposure time (patient-years)	16.31	
Total number of manifestations	33 (2.02)	10 (0.61)
Hypertension	4 (0.25)	1 (0.06)
Hyperuricemia	2 (0.12)	0 (0.00)
Nasopharyngitis	2 (0.12)	0 (0.00)
Edema	2 (0.12)	0 (0.00)
State of consciousness transformation	1 (0.06)	1 (0.06)
Thrombotic microangiopathy	1 (0.06)	1 (0.06)
Bleeding	1 (0.06)	1 (0.06)
Loss of appetite	1 (0.06)	1 (0.06)
Cholelithiasis	1 (0.06)	1 (0.06)
Cholecystitis	1 (0.06)	1 (0.06)
Cerebral infarction	1 (0.06)	1 (0.06)
Pneumonia	1 (0.06)	1 (0.06)
Hives	1 (0.06)	1 (0.06)
βhemolytic streptococcal infection	1 (0.06)	0 (0.00)
Amylase increase	1 (0.06)	0 (0.00)
Gastroesophageal reflux disease	1 (0.06)	0 (0.00)
Arthralgia	1 (0.06)	0 (0.00)
Bronchitis	1 (0.06)	0 (0.00)
Thrombosis	1 (0.06)	0 (0.00)
Eosinophilia	1 (0.06)	0 (0.00)
Dyslipidemia	1 (0.06)	0 (0.00)
Renal dysfunction	1 (0.06)	0 (0.00)
Blister	1 (0.06)	0 (0.00)
Headache	1 (0.06)	0 (0.00)
Fever	1 (0.06)	0 (0.00)
Insomnia	1 (0.06)	0 (0.00)
Sinusitis	1 (0.06)	0 (0.00)

### Characteristics of patients with TMA complicated by underlying disease or complement-amplifying condition (secondary TMA)

This interim analysis included 27 patients with TMA complicated by underlying diseases, which is classified as secondary TMA in Japanese clinical guides 2015 [2] and as secondary aHUS in other reports [8, 14]. Median age (range) at first eculizumab administration was 50 (18–89) years, and median (range) total duration of eculizumab treatment was 2 (0–52) weeks. Complement-related gene variants were examined in eight patients and identified in three patients (37.5%), who were clinically diagnosed as secondary TMA based on underlying diseases or complement amplifying condition, i.e., kidney transplantation and adult-onset Still’s disease (Table 4). Eight patients were treated with eculizumab for < 1 week, 11 patients for ≥ 1 to < 4 weeks, four patients for ≥ 4 to < 26 weeks, and four patients for ≥ 26 weeks. At the date of data cut-off, 26 of 27 patients had discontinued eculizumab therapy because of doctor’s judgement (*n* = 11), due to response observed or symptom improved (*n* = 8), insufficient response to the treatment (*n* = 3), death (*n* = 11), adverse event (*n* = 3), and hospital change (*n* = 1) (Supplementary Table 6).

**Table 4:** Outcome for patients with secondary TMA (*n* = 27)

Underlying disease/complement amplifying condition	No. of patients	Outcome			
		Improvement of symptoms/TMA	Insufficient response	Death	Other/unknown
Kidney transplantation	5	4^a^	–	–	1^b^
Bone marrow transplantation					
Bone marrow transplantation	7	–	–	6 (5^c^)	1^d^
Drug/infection	2	–	–	2 (2^c^)	–
Autoimmune disease	4	1	–	2^e^	1^f^
Infection	1	1	–	–	–
Surgery	1	1	–	–	–
Pancreatitis	1	–	–	1	–
Other/unknown	6	1	1	4^g^ (1^c^)	-

Note that primary diseases of five patients leading to kidney transplantation were reported as chronic glomerulonephritis, lupus nephritis, unknown renal failure (*n* = 1 each), and unknown chronic renal failure (*n* = 2)

^a^*CFH* and *CFB* variants were identified in 1 patient. Identified variants were CFH-p.Val623Ile, p.Glu936Asp and CFB-p.Arg32Trp. CFH-p. Glu936Asp variant is a common variant

^b^Eculizumab treatment was continued. *CFH* and *CFI* variants were identified. (There was no detailed description of variants.)

^c^Patients had malignancy as complications

^d^Improvement by the other treatment was reported

^e^One patient was scleroedema

^f^Outcome was not reported due to hospital change

^g^One patient was adult-onset Still’s disease. *CFH, CFI* and *THBD* variants were identified in the patient. Identified variants were CFH-p.Glu936Asp, CFI-p.Arg406His and THBD-p.Ala473Val. CFH-p.Glu936Asp, CFI-p.Arg406His and THBD-p.Ala473Val variants are common variants

### Outcomes for secondary TMA patients

The investigators noted that the 27 patients with secondary TMA included 14 patients who had undergone transplantation (5 kidney transplants, and 9 bone marrow transplants) and 4 with autoimmune diseases (Table 4).

Notably, improvement of TMA was reported in 4/5 patients who had received kidney transplants: the four patients who showed TMA improvement later discontinued eculizumab; the fifth patient was continuing eculizumab at the date of data cut-off. The improvement of symptoms or TMA was also reported in 4 of 13 patients with autoimmune disease, infection, surgery, pancreatitis and “other” (*n* = 1 each).

Fifteen patients died after discontinuation of eculizumab administration. Fourteen deaths were judged to be unrelated to eculizumab. The other patient is described in the “safety” section below. Among these 15 patients who died, 8 had malignant tumors as complications. Eight patients received only 1 dose of eculizumab and seven of these patients died of severe underlying disease within 2 weeks after starting eculizumab.

### Safety in secondary TMA patients

In the 27 patients with secondary TMA, total exposure time during eculizumab treatment was 4.49 patient-years, and 11 ARs (2.45 per patient-year) were reported in six patients during eculizumab treatment (Table 5). All ARs occurred in 1 patient each (0.22 per patient-year for all). Neither meningococcal infection nor infusion reaction was reported during eculizumab treatment. Eight serious ARs were reported in five patients (0.22 per patient-year); of these, the infection-related serious ARs were pneumonia, pulmonary mycosis, and cytomegalovirus infection (1 patient each).

**Table 5:** Treatment-emergent adverse reaction in patients with secondary TMA (*n* = 27)

	Adverse reaction	Serious adverse reaction
	Number of cases (/person-year)	Number of cases (/person-year)
Total exposure time (patient-years)	4.49	
Total number of manifestations	11 (2.45)	8 (1.78)
Cytomegalovirus infection	1 (0.22)	1 (0.22)
Interstitial lung disease	1 (0.22)	1 (0.22)
Death	1 (0.22)	1 (0.22)
Ventricular flutter	1 (0.22)	1 (0.22)
Multiple organ dysfunction syndrome	1 (0.22)	1 (0.22)
Pneumonia	1 (0.22)	1 (0.22)
Pulmonary mycosis	1 (0.22)	1 (0.22)
Alveolar hemorrhage	1 (0.22)	1 (0.22)
Hypoalbuminemia	1 (0.22)	0 (0.00)
Insomnia	1 (0.22)	0 (0.00)
Delirium	1 (0.22)	0 (0.00)

Death due to an AR occurred in 1 patient with secondary TMA (Table 5); a 27-year-old woman presented a TMA with adult-onset Still’s disease by upper respiratory infections. The patient was treated with eculizumab 11 days after the onset of the TMA and died 2 days after the single dose of eculizumab given. Association between the death and the use of eculizumab could not be confirmed.

## Discussion

This interim analysis provides the first opportunity to present real-world follow-up data on the safety and effectiveness of eculizumab for adult patients with aHUS in Japan. In the current analysis, 68% and 57% of aHUS patients achieved the primary endpoints of TMA event–free status and PLT normalization, respectively. In contrast, TMA event-free was achieved in 88% (trial 1) and 80% (trial 2) of patients with aHUS and PLT normalization was achieved in 82% (trial 1) of patients by week 26 in the study by Legendre et al. [10]. In the other study by Fakhouri et al. [15], these clinical endpoints, TMA event–free status and PLT normalization, were achieved in 90% and 98% of patients by week 26 of eculizumab treatment, respectively. Moreover, a complete TMA response was achieved in 65% (trial 1) and 25% (trial 2) of patients at week 26 in past trials [10], and in about 28% of the patients in this analysis.

In general, patients in clinical trials are usually selected and have less comorbidity than those in real-world analyses; elderly patients, unclear diagnosis and patients with comorbidities are frequently excluded. In clinical trials the treatment duration and dosing is also strictly controlled, while this is up to the judgement of the treating physician in observational real-life registries like this PMS study. Such differences might in part explain the disparity of the outcome between clinical trial and this real-life analysis. In fact, the patients in the current analysis were older (median 58 years) than those in previous trials (median 28 years [10], and mean 40 years [15]). Kidney function was worse in this study (SCr: median 3.67 mg/dL) than in the pivotal trials (SCr: trial 1, median 2.89 mg/dL; trial 2, median 2.64 mg/dL [10]). Moreover, in the current study, 58.6% of patients with aHUS reported a medical history, and differentiation between a HUS and other TMAs may be complex in an adult population.

The LDH improvement is integral parameters of the endpoints. Previous reports indicate that damage to vascular endothelium persists for 30–50 weeks after the first dose of eculizumab [16]. This finding might be a reason of the LDH normalization in less than 60% patients in this study especially under the diverse patient characteristics mentioned above, which could lead to the reduced achievement of complete TMA response.

Prior studies suggest that eculizumab is well tolerated [10, 11]. In this study, each of the ARs was reported in very few patients, although some reactions were classified as serious. Due to the mechanism of action, eculizumab may increase the risk of infection by encapsulated bacterial organisms, particularly *Neisseria meningitidis* [9]. This was closely monitored in the clinical studies and two cases of meningococcal infection were reported from 38 patients with 26 weeks of treatment [15]. In this adult study, none of the 9 infection-related ARs were meningococcal, and no predominant pathogen was detected.

The patient population of the PMS also included those with secondary TMA. A retrospective study has reported on the benefit of eculizumab in “secondary aHUS”, although outcomes were different by specific underlying disease [14]. The Japanese Society of Nephrology has issued precautions for use of eculizumab in patients with secondary TMA [17]. In the present analysis, 15 of 27 patients with secondary TMA died; most of these patients had TMA associated with hematopoietic stem-cell transplantation (HSCT) or cancer. However, among patients with TMA after kidney transplantation, eculizumab treatment resulted in outcomes better than those for other secondary TMAs, which is consistent with previous reports [18]. Interestingly, a rare variant in the CFH or CFI gene in 29% (7/29) of French patients with *de novo* TMA after kidney transplantation was reported [19]. Although genetic examination is necessary to identify the frequency and the variation of complement gene mutations, and the degree of complement’s contribution in the TMA after kidney transplant is still unknown in Japanese patients, the TMA associated with kidney transplant might be considered as a unique TMA different from other secondary TMA.

Similarly, rare variants of complement genes were identified in 40–86% of pregnancy related aHUS, 46% of patients with HELLP syndrome [20–23] and 67% of hypertension associated TMA [24] in previous reports. Moreover, 65% of children and young adult patients with HSCT associated TMA had genetic variants in at least one complement related gene compared with 9% of patients without TMA [25]. Thus, the frequency and the variation of complement gene variant, and the degree of complement’s contribution need to be evaluated in Japanese patient population with the secondary TMA in the future.

Because of the observational design of the PMS, this interim analysis had some limitations, including the absence of a control group, possible underreporting of results and outcomes, missing data, inadequate or incomplete follow-up, and possible variability in the interpretation of disease characteristics and AEs by physicians at different medical institutions. In addition, the clinical practice setting resulted in greater variability in patient background, medical practice and treatment, and follow-up schedule. Therefore, the results should be carefully interpreted.

In conclusion, this interim analysis confirmed the acceptable safety profile and effectiveness of eculizumab for Japanese adult aHUS patients in real-world settings. A subsequent analysis is planned at a later data-cut at the end of the study period to evaluate the long-term safety and effectiveness.

## Electronic supplementary material

Below is the link to the electronic supplementary material.

Supplementary material 1 (DOCX 59 KB)

Supplementary material 2 (PPTX 849 KB)
