# Supplementary material for: Safety and effectiveness of eculizumab for adult patients with atypical hemolytic–uremic syndrome in Japan: interim analysis of post-marketing surveillance
Source: Clin Exp Nephrol. 2018 Jun 29;23(1):65–75. doi: 10.1007/s10157-018-1609-8 (PMC6344388; doi:10.1007/s10157-018-1609-8)
Supplement: Supplementary file 1 — Supplementary material 1 (DOCX 59 KB) [file 10157_2018_1609_MOESM1_ESM.docx]

**Supplementary material**

Supplementary Fig. 1 Kaplan-Meier survival curve for aHUS (complement-mediated HUS) patients

Survival probability was evaluated in 29 aHUS patients. The number of patients at risk are shown below the X-axis label.

Supplementary Fig. 2 Change in platelet count (a), LDH (b), and serum Creatinine (c) for individual aHUS (complement-mediated HUS) patients

**Supplementary References**

1 Matsumoto T, Fan X, Ishikawa E, Ito M, Amano K, Toyoda H et al., Analysis of patients with atypical hemolytic uremic syndrome treated at the Mie University Hospital: concentration of C3 p.I1157T mutation. Int J Hematol. 2014; 100: 437-42.

2 Manuelian T, Hellwage J, Meri S, Caprioli J, Noris M, Heinen S et al., Mutations in factor H reduce binding affinity to C3b and heparin and surface attachment to endothelial cells in hemolytic uremic syndrome. J Clin Invest. 2003; 111: 1181-90.
