# Supplementary material for: Safety and effectiveness of eculizumab for adult patients with atypical hemolytic–uremic syndrome in Japan: interim analysis of post-marketing surveillance
Source: Clin Exp Nephrol. 2018 Jun 29;23(1):65–75. doi: 10.1007/s10157-018-1609-8 (PMC6344388; doi:10.1007/s10157-018-1609-8)
Supplement: Supplementary file 2 — Supplementary material 2 (PPTX 849 KB) [file 10157_2018_1609_MOESM2_ESM.pptx]

## Slide 1
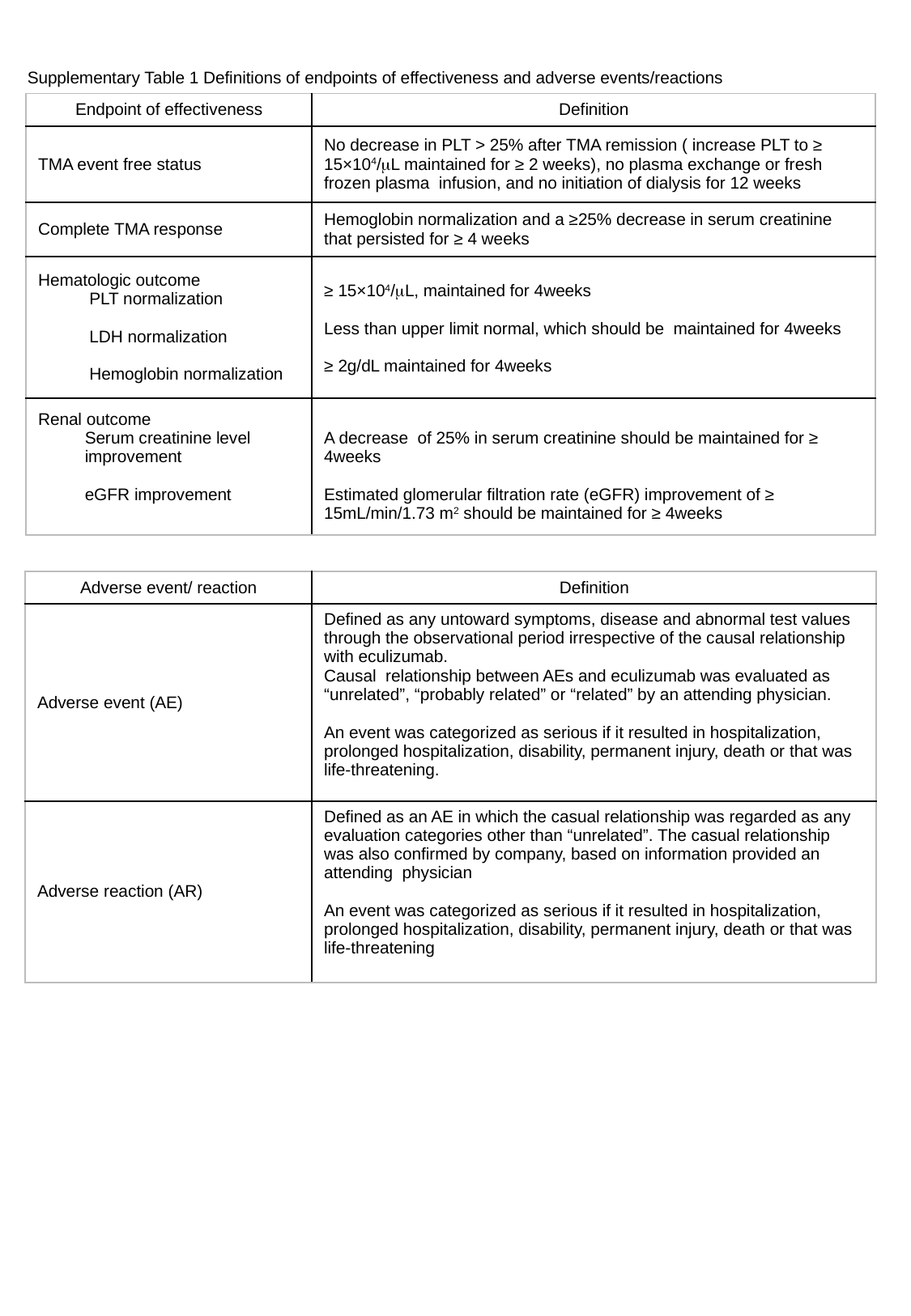

Supplementary Table 1 Definitions of endpoints of effectiveness and adverse events/reactions
| Endpoint of effectiveness | Definition |
| --- | --- |
| TMA event free status | No decrease in PLT > 25% after TMA remission ( increase PLT to ≥ 15×104/mL maintained for ≥ 2 weeks), no plasma exchange or fresh frozen plasma infusion, and no initiation of dialysis for 12 weeks |
| Complete TMA response | Hemoglobin normalization and a ≥25% decrease in serum creatinine that persisted for ≥ 4 weeks |
| Hematologic outcome PLT normalization LDH normalization Hemoglobin normalization | ≥ 15×104/mL, maintained for 4weeks Less than upper limit normal, which should be maintained for 4weeks ≥ 2g/dL maintained for 4weeks |
| Renal outcome Serum creatinine level improvement eGFR improvement | A decrease of 25% in serum creatinine should be maintained for ≥ 4weeks Estimated glomerular filtration rate (eGFR) improvement of ≥ 15mL/min/1.73 m2 should be maintained for ≥ 4weeks |
| Adverse event/ reaction | Definition |
| --- | --- |
| Adverse event (AE) | Defined as any untoward symptoms, disease and abnormal test values through the observational period irrespective of the causal relationship with eculizumab. Causal relationship between AEs and eculizumab was evaluated as “unrelated”, “probably related” or “related” by an attending physician. An event was categorized as serious if it resulted in hospitalization, prolonged hospitalization, disability, permanent injury, death or that was life-threatening. |
| Adverse reaction (AR) | Defined as an AE in which the casual relationship was regarded as any evaluation categories other than “unrelated”. The casual relationship was also confirmed by company, based on information provided an attending physician An event was categorized as serious if it resulted in hospitalization, prolonged hospitalization, disability, permanent injury, death or that was life-threatening |

## Slide 2
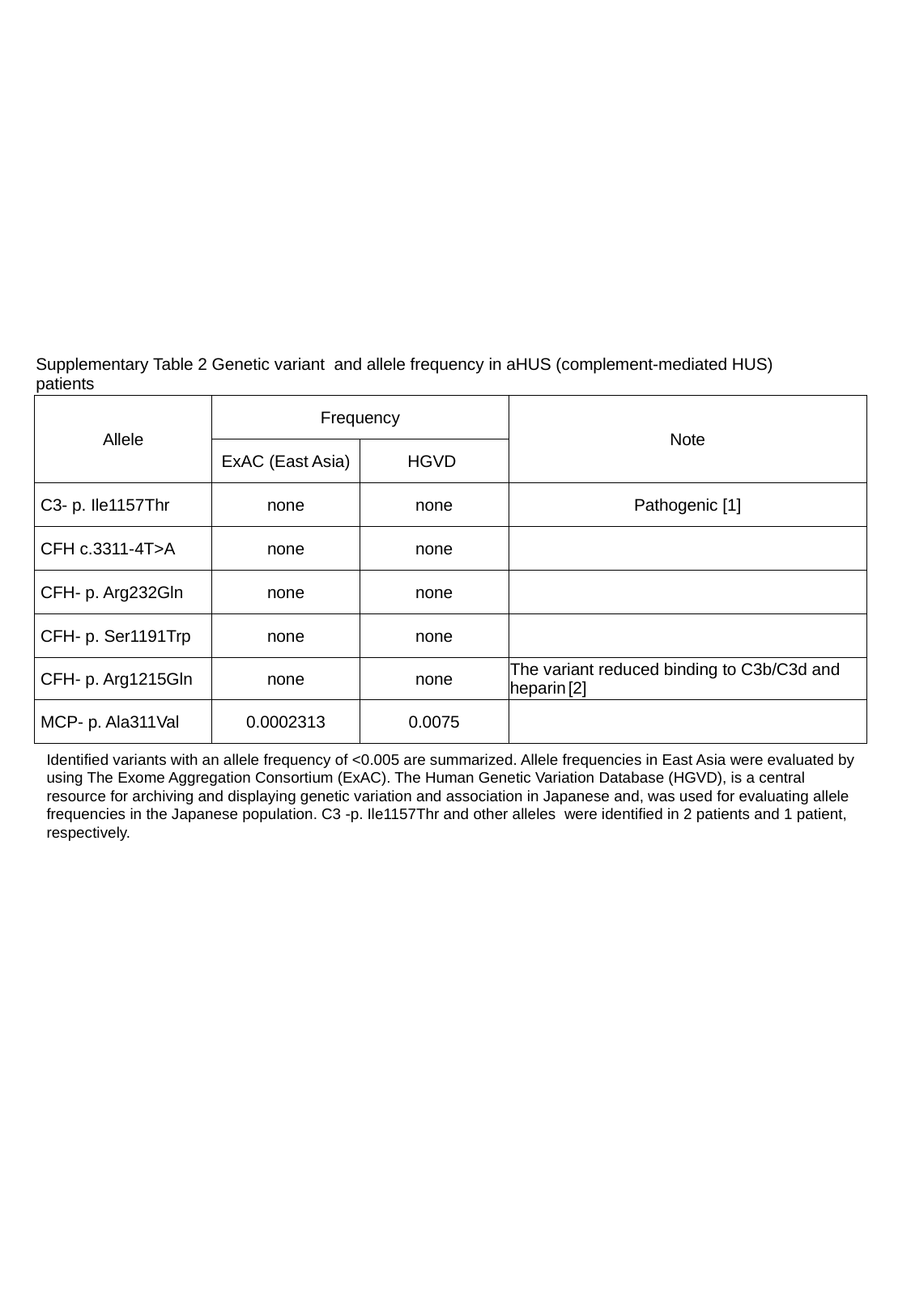

| Supplementary Table 2 Genetic variant and allele frequency in aHUS (complement-mediated HUS) patients | | | |
| --- | --- | --- | --- |
| Allele | Frequency | | Note |
| | ExAC (East Asia) | HGVD | |
| C3- p. Ile1157Thr | none | none | Pathogenic [1] |
| CFH c.3311-4T>A | none | none | |
| CFH- p. Arg232Gln | none | none | |
| CFH- p. Ser1191Trp | none | none | |
| CFH- p. Arg1215Gln | none | none | The variant reduced binding to C3b/C3d and heparin [2] |
| MCP- p. Ala311Val | 0.0002313 | 0.0075 | |
Identified variants with an allele frequency of <0.005 are summarized. Allele frequencies in East Asia were evaluated by using The Exome Aggregation Consortium (ExAC). The Human Genetic Variation Database (HGVD), is a central resource for archiving and displaying genetic variation and association in Japanese and, was used for evaluating allele frequencies in the Japanese population. C3 -p. Ile1157Thr and other alleles were identified in 2 patients and 1 patient, respectively.

## Slide 3
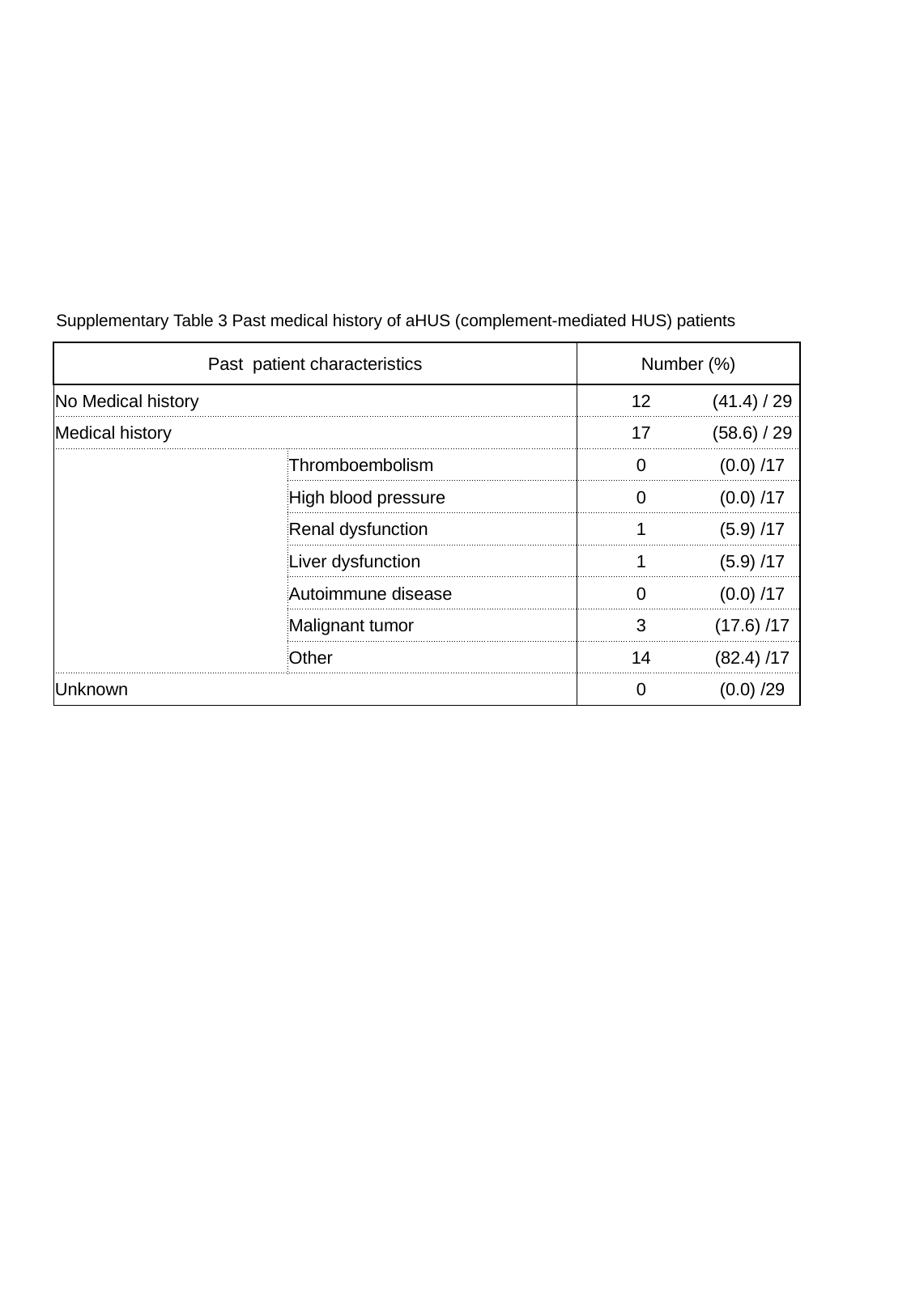

Supplementary Table 3 Past medical history of aHUS (complement-mediated HUS) patients
| Past patient characteristics | | Number (%) | |
| --- | --- | --- | --- |
| No Medical history | | 12 | (41.4) / 29 |
| Medical history | | 17 | (58.6) / 29 |
| | Thromboembolism | 0 | (0.0) /17 |
| | High blood pressure | 0 | (0.0) /17 |
| | Renal dysfunction | 1 | (5.9) /17 |
| | Liver dysfunction | 1 | (5.9) /17 |
| | Autoimmune disease | 0 | (0.0) /17 |
| | Malignant tumor | 3 | (17.6) /17 |
| | Other | 14 | (82.4) /17 |
| Unknown | | 0 | (0.0) /29 |

## Slide 4
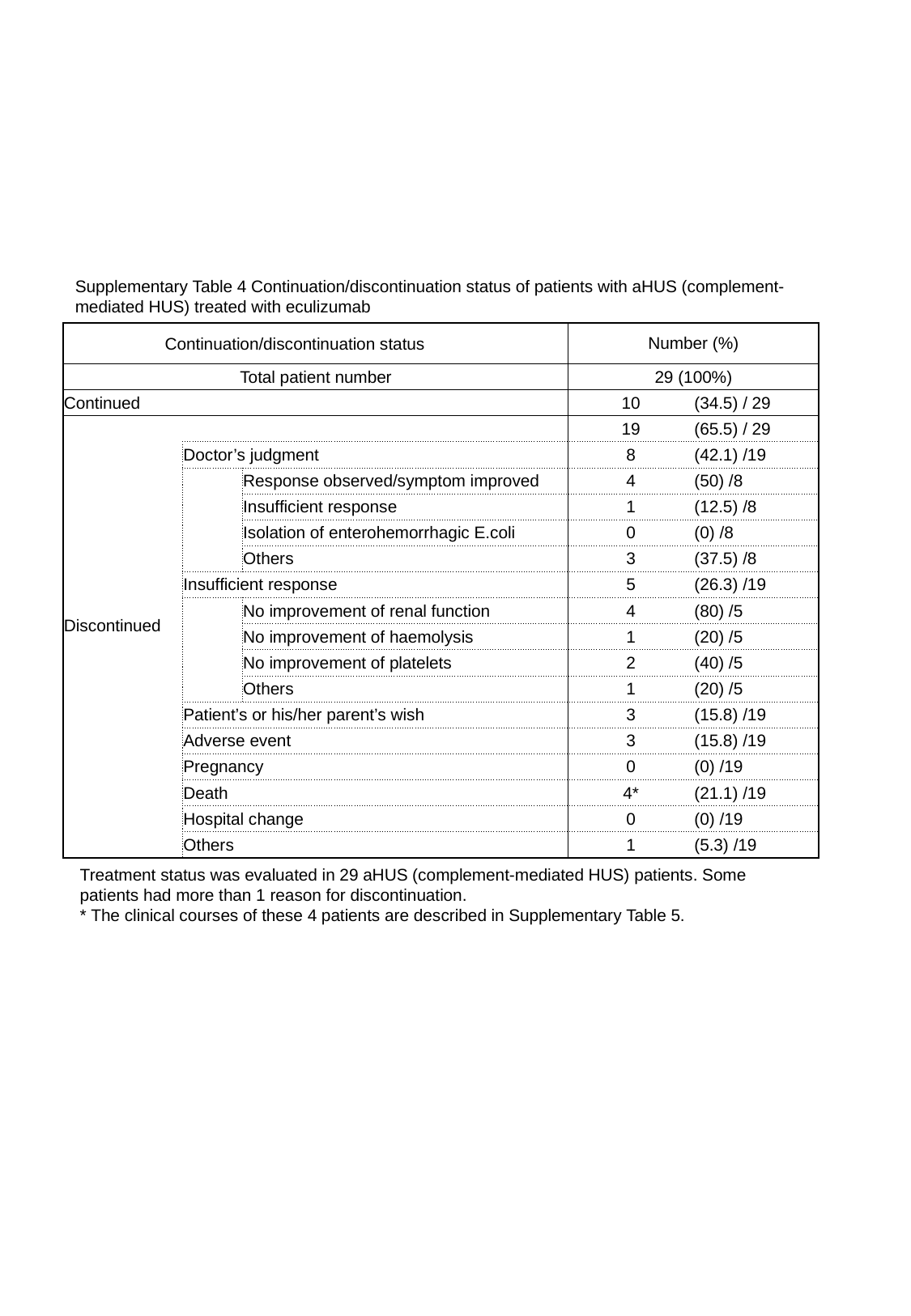

Supplementary Table 4 Continuation/discontinuation status of patients with aHUS (complement-mediated HUS) treated with eculizumab
| Continuation/discontinuation status | | | | Number (%) | |
| --- | --- | --- | --- | --- | --- |
| Total patient number | | | | 29 (100%) | |
| Continued | | | | 10 | (34.5) / 29 |
| Discontinued | | | | 19 | (65.5) / 29 |
| | Doctor’s judgment | | | 8 | (42.1) /19 |
| | | Response observed/symptom improved | | 4 | (50) /8 |
| | | Insufficient response | | 1 | (12.5) /8 |
| | | Isolation of enterohemorrhagic E.coli | | 0 | (0) /8 |
| | | Others | | 3 | (37.5) /8 |
| | Insufficient response | | | 5 | (26.3) /19 |
| | | No improvement of renal function | | 4 | (80) /5 |
| | | No improvement of haemolysis | | 1 | (20) /5 |
| | | No improvement of platelets | | 2 | (40) /5 |
| | | Others | | 1 | (20) /5 |
| | Patient’s or his/her parent’s wish | | | 3 | (15.8) /19 |
| | Adverse event | | | 3 | (15.8) /19 |
| | Pregnancy | | | 0 | (0) /19 |
| | Death | | | 4\* | (21.1) /19 |
| | Hospital change | | | 0 | (0) /19 |
| | Others | | | 1 | (5.3) /19 |
Treatment status was evaluated in 29 aHUS (complement-mediated HUS) patients. Some patients had more than 1 reason for discontinuation.
* The clinical courses of these 4 patients are described in Supplementary Table 5.

## Slide 5
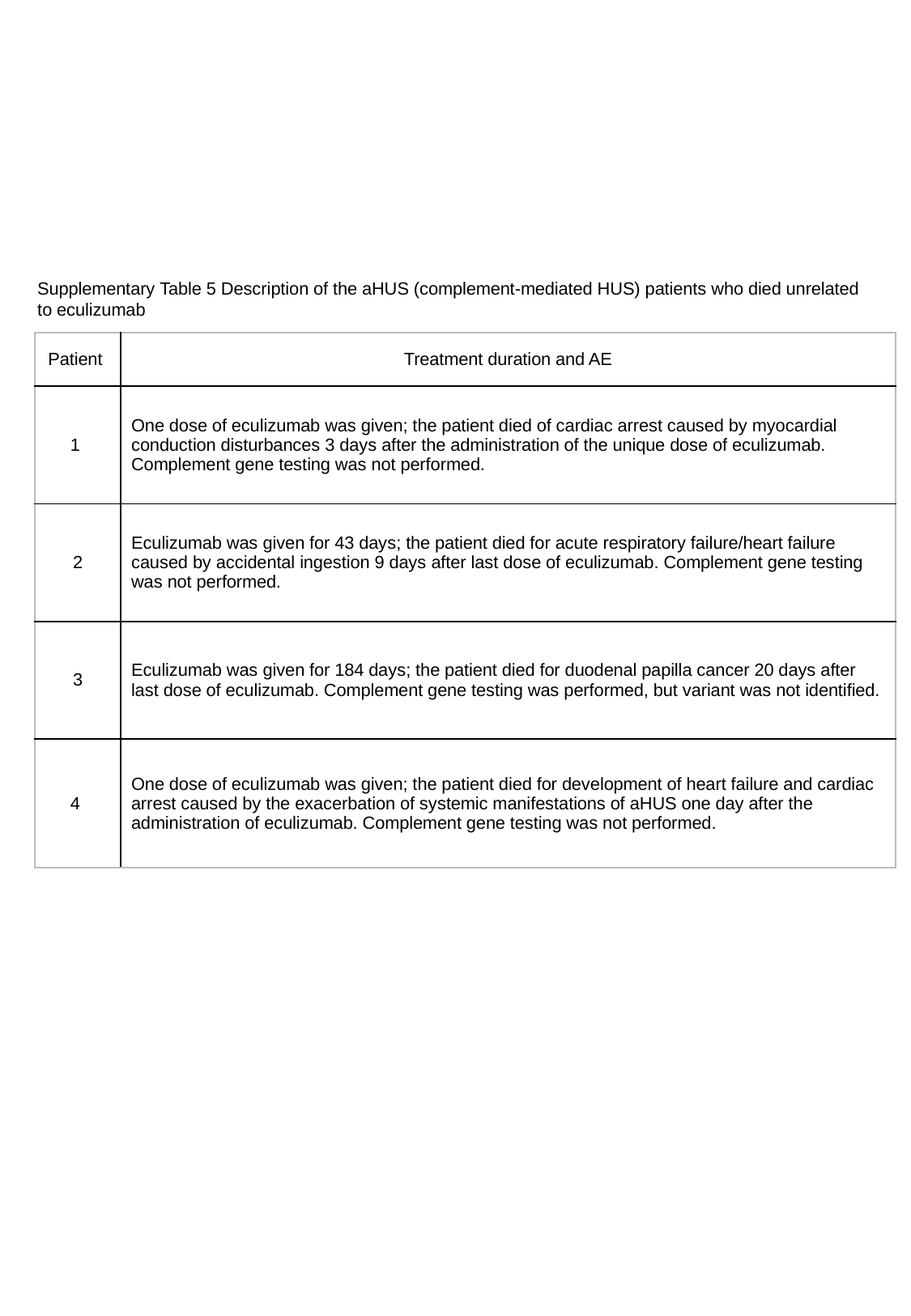

Supplementary Table 5 Description of the aHUS (complement-mediated HUS) patients who died unrelated to eculizumab
| Patient | Treatment duration and AE |
| --- | --- |
| 1 | One dose of eculizumab was given; the patient died of cardiac arrest caused by myocardial conduction disturbances 3 days after the administration of the unique dose of eculizumab. Complement gene testing was not performed. |
| 2 | Eculizumab was given for 43 days; the patient died for acute respiratory failure/heart failure caused by accidental ingestion 9 days after last dose of eculizumab. Complement gene testing was not performed. |
| 3 | Eculizumab was given for 184 days; the patient died for duodenal papilla cancer 20 days after last dose of eculizumab. Complement gene testing was performed, but variant was not identified. |
| 4 | One dose of eculizumab was given; the patient died for development of heart failure and cardiac arrest caused by the exacerbation of systemic manifestations of aHUS one day after the administration of eculizumab. Complement gene testing was not performed. |

## Slide 6
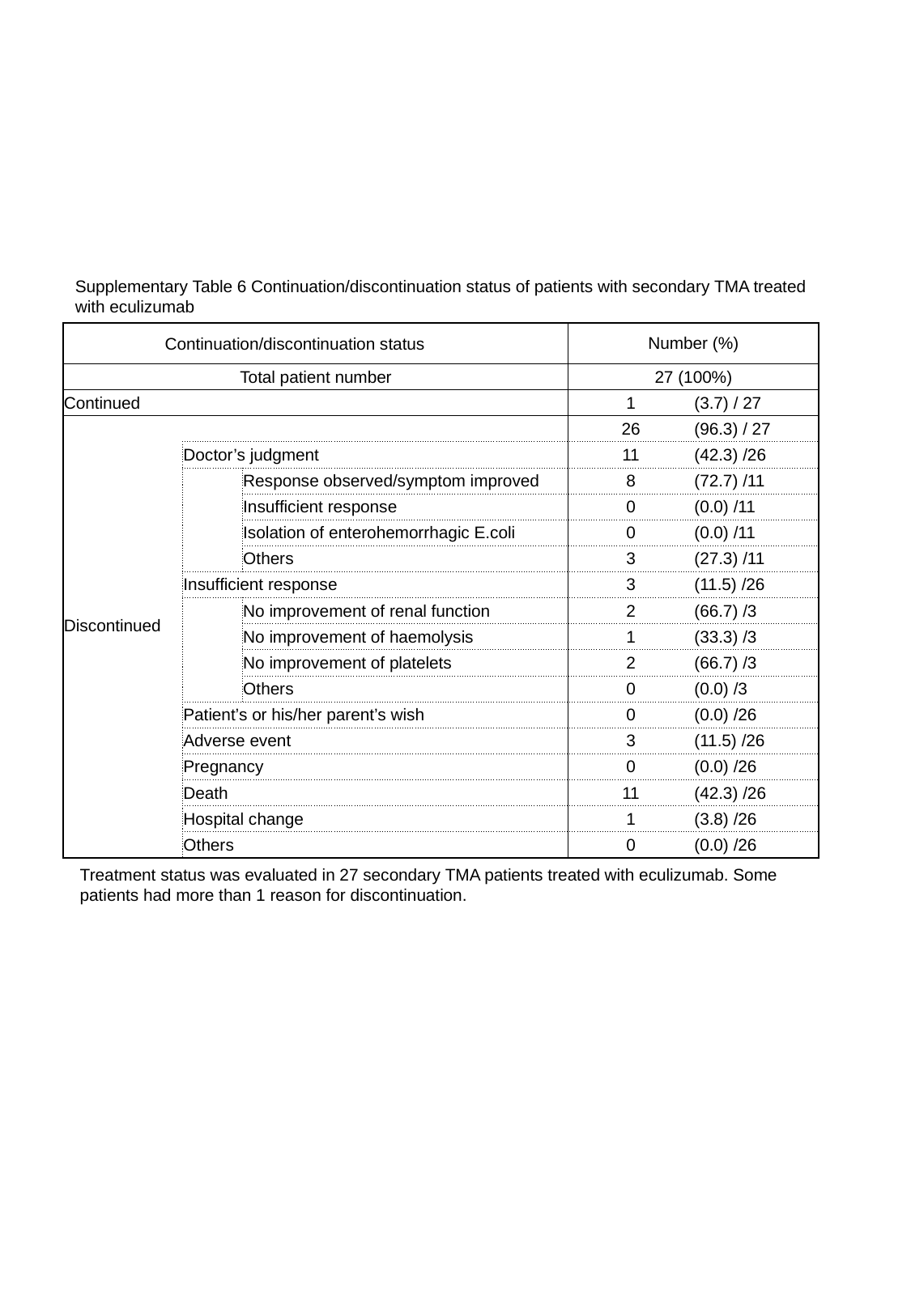

Supplementary Table 6 Continuation/discontinuation status of patients with secondary TMA treated with eculizumab
| Continuation/discontinuation status | | | | Number (%) | |
| --- | --- | --- | --- | --- | --- |
| Total patient number | | | | 27 (100%) | |
| Continued | | | | 1 | (3.7) / 27 |
| Discontinued | | | | 26 | (96.3) / 27 |
| | Doctor’s judgment | | | 11 | (42.3) /26 |
| | | Response observed/symptom improved | | 8 | (72.7) /11 |
| | | Insufficient response | | 0 | (0.0) /11 |
| | | Isolation of enterohemorrhagic E.coli | | 0 | (0.0) /11 |
| | | Others | | 3 | (27.3) /11 |
| | Insufficient response | | | 3 | (11.5) /26 |
| | | No improvement of renal function | | 2 | (66.7) /3 |
| | | No improvement of haemolysis | | 1 | (33.3) /3 |
| | | No improvement of platelets | | 2 | (66.7) /3 |
| | | Others | | 0 | (0.0) /3 |
| | Patient’s or his/her parent’s wish | | | 0 | (0.0) /26 |
| | Adverse event | | | 3 | (11.5) /26 |
| | Pregnancy | | | 0 | (0.0) /26 |
| | Death | | | 11 | (42.3) /26 |
| | Hospital change | | | 1 | (3.8) /26 |
| | Others | | | 0 | (0.0) /26 |
Treatment status was evaluated in 27 secondary TMA patients treated with eculizumab. Some patients had more than 1 reason for discontinuation.

## Slide 7
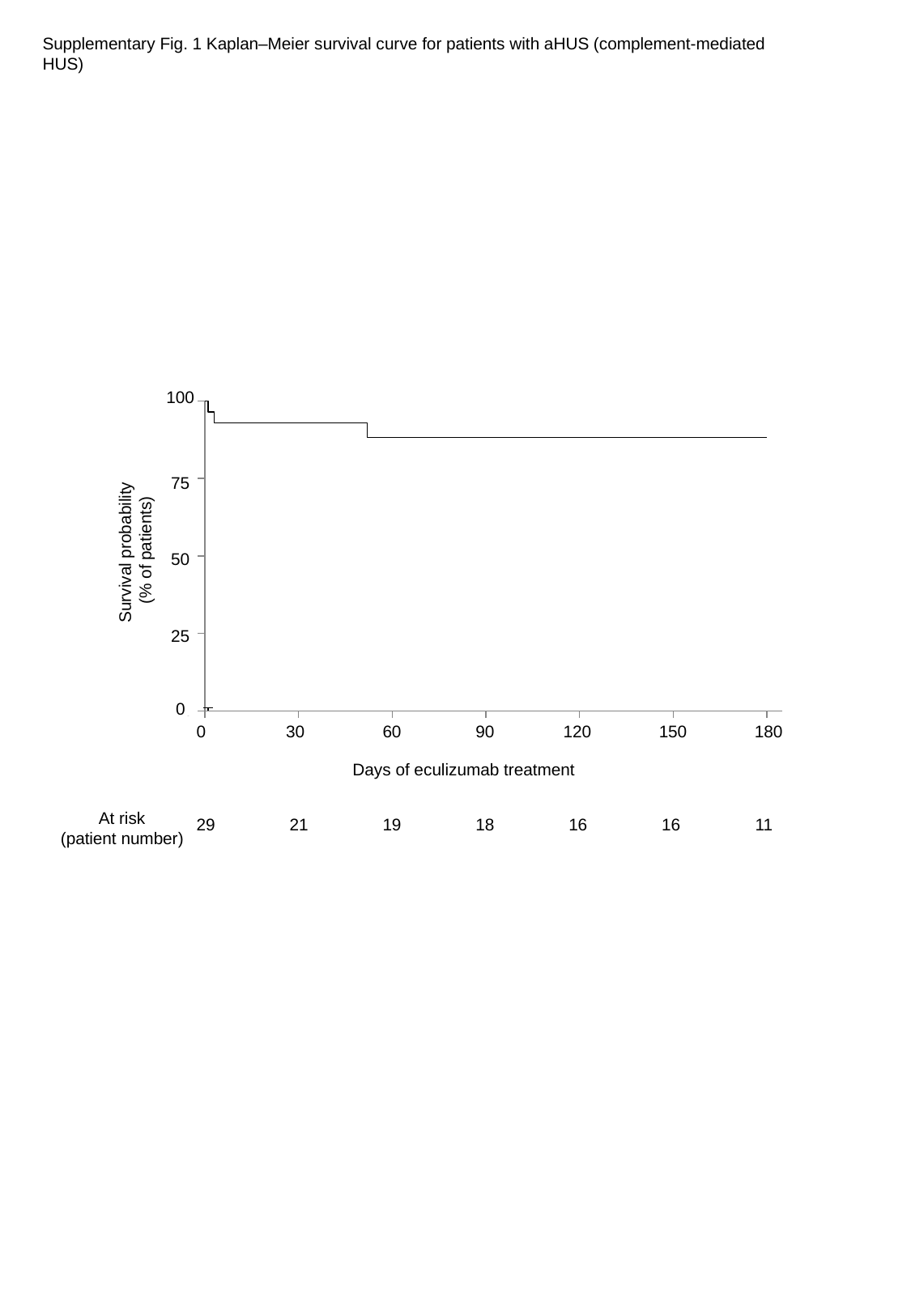

Supplementary Fig. 1 Kaplan–Meier survival curve for patients with aHUS (complement-mediated HUS)
### Chart
| Category | | 成人(初回投与時18歳以上) | | | 0：イベント発現
1：打ち切り(成人) | | |
|---|---|---|---|---|---|---|---|100
75
Survival probability
 (% of patients)
50
25
0
0
30
60
90
120
150
180
Days of eculizumab treatment
At risk
(patient number)
29
21
19
18
16
16
11

## Slide 8
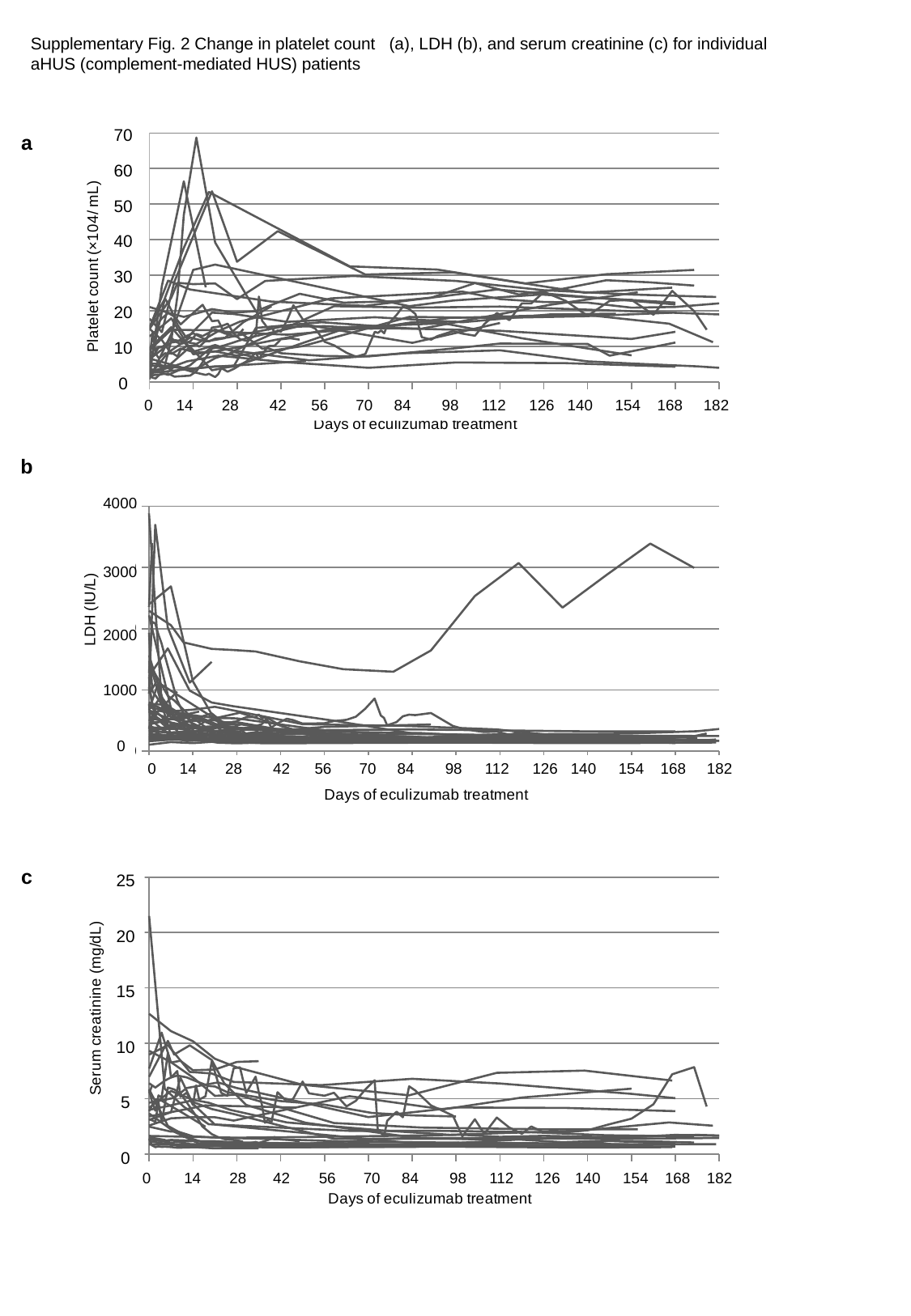

Supplementary Fig. 2 Change in platelet count (a), LDH (b), and serum creatinine (c) for individual aHUS (complement-mediated HUS) patients
### Chart
| Category | 測定値 |
|---|---|70
a
60
50
40
30
20
10
0
0
14
28
42
56
70
84
98
112
126
140
154
168
182
b
### Chart
| Category | 測定値 |
|---|---|4000
3000
2000
1000
0
0
14
28
42
56
70
84
98
112
126
140
154
168
182
### Chart
| Category | 測定値 |
|---|---|
c
25
20
15
10
5
0
0
14
28
42
56
70
84
98
112
126
140
154
168
182
